# Antimycobacterial Activity of a New Peptide Polydim-I Isolated from Neotropical Social Wasp *Polybia dimorpha*

**DOI:** 10.1371/journal.pone.0149729

**Published:** 2016-03-01

**Authors:** Rogerio Coutinho das Neves, Monalisa Martins Trentini, Juliana de Castro e Silva, Karina Smidt Simon, Anamelia Lorenzetti Bocca, Luciano Paulino Silva, Marcia Renata Mortari, Andre Kipnis, Ana Paula Junqueira-Kipnis

**Affiliations:** 1 Department of Microbiology, Immunology, Parasitology and Pathology, Institute of Tropical Pathology and Public Health, Federal University of Goiás, Goiânia, Brazil; 2 Laboratório de Toxinologia. Campus Darcy Ribeiro, Instituto de Ciências Biológicas, Departamento de Ciências Fisiológicas, University of Brasilia, Brasília, Brazil; 3 Depto Biologia Celular. Instituto de Biologia. Laboratório de Imunologia Aplicada, University of Brasilia, Brasília, Brazil; French National Centre for Scientific Research, FRANCE

## Abstract

*Mycobacterium abscessus* subsp. *massiliense*, a rapidly growing mycobacteria (RGM) that is becoming increasingly important among human infectious diseases, is virulent and pathogenic and presents intrinsic resistance to several antimicrobial drugs that might hamper their elimination. Therefore, the identification of new drugs to improve the current treatment or lower the risk of inducing resistance is urgently needed. Wasp venom primarily comprises peptides that are responsible for most of the biological activities in this poison. Here, a novel peptide Polydim-I, from *Polybia dimorpha* Neotropical wasp, was explored as an antimycobacterial agent. Polydim-I provoked cell wall disruption and exhibited non-cytotoxicity towards mammalian cells. Polydim-I treatment of macrophages infected with different *M*. *abscessus* subsp. *massiliense* strains reduced 40 to 50% of the bacterial load. Additionally, the Polydim-I treatment of highly susceptible mice intravenously infected with *M*. *abscessus* subsp. *massiliense* induced 0.8 to 1 log reduction of the bacterial load in the lungs, spleen, and liver. In conclusion, this is the first study to show the therapeutic potential of a peptide derived from wasp venom in treating mycobacteria infections. Polydim-I acts on the *M*. *abscessus* subsp. *massiliense* cell wall and reduce 40–90% of the bacterial load both *in vitro* and *in vivo*. The presented results encourage further studies on the use of Polydim-I as one of the components for *M*. *abscessus* subsp. *massiliense* treatment.

## Introduction

*Mycobacterium abscessus* subsp. *massiliense* is a rapid growing mycobacteria (RGM) that is becoming increasingly important among human infectious diseases and is associated with soft tissue infections that are caused by contaminated hospital equipment and solutions following small invasive procedures or intramuscular injections [1, 2]. This group includes the formally known *Mycobacterium massiliense* [3, 4] that is virulent and pathogenic, inducing a chronic and disseminated evolution in the host, independent of the immune status [3–7]. In Brazil, 2,000 new cases of RGM infections have been diagnosed since 1998, most of which occurred following surgical procedures such as myopia correction, mesotherapic procedures, breast implants, or esthetical procedures, usually associated with contaminated videoscopy equipment and solutions. Among the RGM detected, most of these cases were infections with *M*. *abscessus* subsp. *massiliense* [8, 9].

*M*. *abscessus* subsp. *massiliense* infection outbreaks might be a consequence of the intrinsic resistance of these bacteria to 2% glutaraldehyde, a commonly used high-level disinfecting solution [2]. The most appropriate treatment for *M*. *abscessus* subsp. *massiliense* infections is to use a combination of drugs to avoid the development of resistance. Currently, the most commonly used chemotherapy includes the use of clarithromycin (CLR) together with amikacin (AMK) [5], moxifloxacin (MXF) or cefoxitin (FOX) [6]. The major problem of chemotherapy, in addition to intrinsic resistance, is the development of resistant strains that vary from 4.5% resistance to CLR to 18% to FOX [10]. In addition, these drugs have hepatotoxic, enterotoxic, nephrotoxic, hematotoxic and skin side effects when used for long periods of time, and these side effects may result in non-adherence to therapy [11]. Consequently, new, improved and efficient drugs against RGM are essential.

An interesting approach for the development of new drugs is the use of antimicrobial peptides (AMP), also known as natural antibiotics. Interestingly, AMP can be administered individually or in association with conventional chemotherapeutic drugs and have presented promising results for treating several infectious diseases [12–14]. The mechanisms underlying the activity of AMP actions seem to vary according to each family of molecules, but in general, these activities rely on their ability to permeabilize biological membranes, create pores, or affect intracellular activities [15].

Wasp venoms primarily comprise peptides, including AMP, which are responsible for most of the biological activities of this poison. Among those peptides, the most recurrent are those from the class of mastoparans [16–18], small cationic and amphiphilic alpha-helical peptides that possess high affinity to negatively charged biological membranes. Thus, the venom of social wasps has great potential for the study of new antimicrobials, particularly with respect to antimycobacterial activity. Currently, countless peptides remain unknown because of a lack of studies focusing on the identification of antimicrobial peptides from the venom of Neotropical social wasps. Thus, the aim of this study was to investigate the effects of a novel peptide: Polydim-I that was isolated from the venom of the Neotropical wasp *Polybia dimorpha* as a potential antimycobacterial agent.

## Materials and Methods

### Collection of specimens and venom extraction

Females of *Polybia dimorpha* were collected at Brasília, Distrito Federal, Brazil, under license in accordance with the Normative Instruction No. 154, from March 2007, of IBAMA (Brazilian Institute of Environment and Renewable Natural Resources, license number 21723–1). Moreover, authorization of the Access and Remittance of the Brazilian genetic patrimony was obtained from the CNPq (license number 010476/2013-0). The wasps were sacrificed after freezing at -20°C. Following species identification (Prof. Fernando B. Noll, UNESP-SP), the venom sacs were dissected and macerated in a 10% acetonitrile in deionized water solution and centrifuged at 5000 × *g* for 10 min, at 4°C. The supernatant was carefully collected and submitted to a filtration using an ultra-filter (Millipore) with a 3-kDa cut-off for 30 min at 5000 × *g*. The resultant ultrafiltrate, which was characterized by the presence of low molecular mass compounds, was collected, lyophilized and weighed.

### Peptide fractionation and purification

The filtered extract was resuspended in 2% acetonitrile in water (ACN/H_2_O) and 0.07% trifluoroacetic acid (TFA). The solution was subjected to chromatography using a reversed-phase high performance liquid chromatography (RP-HPLC) column (C_18_ ODS, Jupiter 15 μm, 20 × 250 mm, Phenomenex, Torrence, CA, USA) at a flow rate of 1.5 mL/min and was then eluted using a linear gradient from 5% ACN/H_2_O (v/v) (containing 0.07% TFA) for 20 min, followed by 5 to 60% for 40 min and under 60% for 20 min. The absorbance of the eluted compounds was monitored at 214 and 280 nm, and the fractions were manually collected and subsequently lyophilized.

### MALDI-TOF mass spectrometry

The fractions containing Polydim-I were subjected to identification (purity and identity) through matrix-assisted laser desorption/ionization mass spectrometry time-of flight (MALDI-TOF TOF) (UltraFlex III, Bruker Daltonics, Germany) under reflector (MS) and LIFT^TM^ (MS/MS) positive modes. Prior to analysis, the fractions were dissolved in a saturated solution of α-cyano-4-hydroxycinnamic acid matrix in acetonitrile/water/3% trifluoroacetic acid (5/4/1). Additionally, the peptides’ mass spectra and sequencing were manually interpreted using the FlexAnalysis 3.0 software (Bruker Daltonics, Germany). The monoisotopic molecular mass of the peptide was determined as the ratio between the m/z peaks in the spread profile (m/z ratio from 600 to 3,000). Moreover, similarity searches were performed using Fasta3 programs, the Expasy12 server and BLASTP13.

### Polydim-I synthesis

The Polydim-I (a novel peptide derived from wasp *Polybia dimorpha* venon) was synthesized through solid phase chromatography using an N-9-fluorophenylmethoxy-carbonyl (F-MOC) strategy and purified through RP-HPLC at Aminotech Development and Technology. Moreover, the purity and sequence of the peptide were assessed using the same protocol described for the identification of the natural peptide.

### Bacterial strain preparation

*M*. *abscessus* subsp. *massiliense* isolates GO 01, GO 06, GO 07, GO 08, GO 13, and GO 18, were randomly chosen from a collection bank of isolates from a Brazilian epidemic outbreak that occurred in Goiânia, State of Goiás during 2005 and 2007 [5], and CRM0020 (*M*. *abscessus* subsp. *massiliense*), and ATCC19977 (*M*. *abscessus* subsp. *abscessus*) reference strains were included. *M*. *abscessus* subsp. *massiliense* were identified previously by colony morphology, biochemical testes, PCR-Restriction-enzyme Analysis (PRA-hsp65), and partial rpoB gene sequencing [5]. For the *in vitro* experiments, aliquots of frozen mycobacteria were grown in Mueller Hinton (MH, HIMEDIA) with 0.05% Tween 80 at 35°C with agitation (200 × rpm) for three days. The culture concentration was adjusted to a 0.5 scale of McFarland (approximately 1.5 × 10^8^ CFU/mL) after vigorous homogenization to dissolve clumps.

For mice intravenous injection, *M*. *abscessus* subsp. *massiliense* GO 06, previously evaluated in mice [19] with known concentration and maintained at -80°C were thawed and suspended in phosphate-buffered saline (PBS), 0.05% Tween 80 to approximately 10^6^ CFU/mL.

All *M*. *abscessus* subsp. *massiliense* suspensions were plated onto MH agar for CFU determination.

### Minimum inhibitory concentration test (MIC)

MIC was determined according to ATS/IDSA recommendations [20]. Bacterial cultures were further adjusted, and 100 CFU was deposited into each well of a 96-well microplate that contained serially diluted Polydim-I in levels ranging from 243.2 to 3.8 μg/ml. Lyophilized Polydim-I was resuspended in 3% DMSO and further diluted in PBS before adding to the cultures. The control wells received bacteria and PBS (growth control) or CLR at concentrations ranging from 8 to 0.06 μg/mL (inhibition control). The plates were incubated for three days at 35°C. The MIC (lowest concentration without bacterial growth) was estimated by CFU counting after plating in MH media.

### Scanning Electron Microscopy analysis of peptide effect on *M*. *abscessus* subsp. *massiliense*

*M*. *abscessus* subsp. *massiliense* colonies grown on MH agar were removed from the plates and exposed to 3.8 or 7.6 μg/mL of Polydim-I or 0.5 μg/mL of clarithromycin for 24 h. As a negative control, the colonies were incubated with PBS alone. After incubation, the cells were fixed with modified Karnovsky solution (paraformaldehyde 1% and glutaraldehyde 3% in 0.07 M cacodylate buffer, pH 7.2) for 30 min at 4°C. The fixative solution was subsequently removed, and the samples were dehydrated through increasing concentrations of ethanol solution (30%, 50%, 70%, 90%, and 100%) for 10 min, followed by acetone and hexamethyldisilazane (HMDS) (V/V) for an additional 5 min. The final dehydration step was performed with HMDS for 5 min. After the samples were completely dry, a thin layer of gold was deposited using a Denton Vacuum Desk V apparatus. The images were obtained using a Jeol JSM – 6610 microscope (Jeol, Japan) equipped with energy dispersive spectroscopy—EDS (Thermo scientific NSS Spectral Imaging).

### Animals

BALB/c and IFN-γKO (Knockout) mice were obtained from the Instituto de Patologia Tropical e Saúde Pública from Federal University of Goiás animal facilities. All animals were housed according to the Colegio Brasileiro de experimentação animal (COBEA) under the supervision of a Veterinarian. Female mice that were 6–8 weeks old and weighed 22–25 g from both lineages were used. Five groups of 6 animals (IFN-γKO) each were maintained in a HEPA filtered rack with water and food available *ad libitum*. To enrich the animal environment, cardboard tubes were weekly added to the cages for the 28–30 days duration of the study. Ten BALB/c mice that were maintained under the same conditions were used to obtain macrophages. All protocols were submitted and approved through the Comitê de Ética no Uso de Animais em Pesquisa-UFG (Protocol: 027/14). All experiments were repeated three times, and a total of 10 BALB/c and 90 IFN-γKO mice were used.

### Peritoneal macrophages cultures

BALB/c mice were injected with thioglycolate 72h before the peritoneal macrophages were collected. The macrophages (1 × 10^6^ cells/ml), which were diluted in RPMI-1640 (Sigma, St. Louis, USA) supplemented with penicillin/streptomycin (100 U/mL, 100 g/mL), 2 mM L-glutamine, 2 mM non-essential amino acids, 1 mM sodium pyruvate (all reagents from Sigma-Aldrich, St Louis, MO, USA) and 10% fetal bovine serum (FBS), were incubated for 24 h at 37°C, 5% CO_2_, in 24-well microplates containing 18-mm circular cover slides or in 96 well microplates. Peritoneal macrophages plated in 24-well microplate were used to evaluate the morphology/viability after Polydim-I treatment of infected macrophages. The peritoneal macrophages plated in 96 wells plates were used to address the microbicidal effects of Polydim-I on infected macrophages (see details bellow).

### J774 macrophage cytotoxicity evaluation

Cytotoxic activity was measured using J774 macrophages cells treated with 7.6, 15.2, 60.8, and 121.6 μg/mL of the peptide for 24 h under the same conditions as described for peritoneal macrophages. The percentage of cytotoxicity of Polydim-I was evaluated by lactate dehydrogenase (LDH) release assay using Cyto Tox 96 kit (Promega).

### Hemolysis

A solution of fresh human red blood cells was used to test the hemolytic capacity of the peptide at levels ranging from 7.6 to 121.6 μg/mL. Fifty micro liters of a 3% cell suspension of EDTA treated blood obtained from healthy volunteers were added to a 96 well microplate. Different concentrations of the peptide (v/v) were added to each well and after one hour incubation, the microplates were centrifuged, and the supernatant was carefully collected and read at 540 nm using an ELISA microplate reader (Thermo Labsystems, USA). As positive lysis control the erythrocytes were incubated with water. The percentage of hemolysis from each test was calculated relative to the positive control. Blood were collected from healthy individuals that signed a written Informed Consent. The procedures and the informed consent were approved by the Ethics Committe on Human Research of Faculty of Medicine/University of Brasilia (UNB).

### Mycobactericidal activity of Polydim-I on infected macrophages

Peritoneal macrophages that were obtained as described above were infected with *M*. *abscessus* subsp. *massiliense* (MOI 10:1) for three hours. Subsequently, the cultures were washed three times with warm RPMI. The obtained supernatants from the washes were pooled and plated onto MH agar for CFU determination. The infected macrophages were treated with Polydim-I at 7.6 μg/mL in 200 μL of RPMI. After three days of incubation, the supernatant was removed, the cells were washed three times with warm PBS to remove extra cellular bacteria and the cells were lysed with water. The resulting lysate and supernatant were spread onto MH agar for CFU determination. To estimate the number of bacteria phagocytosed by macrophages prior to Polydim-I treatment, the number of CFU obtained from the pooled supernatant after three hours of infection was subtracted from the total inoculum (10^6^). Additionally, infected peritoneal macrophages were cultivated in 24 well plate containing circular cover slides to investigate the cell morphology. Three independent experiments were performed. Additionally, two experiments were performed using non-activated peritoneal macrophages to confirm Polydim-I bactericidal activity.

The cover slides from the 24-well microplates were washed with PBS and fixed with methanol for 5 min. Then, the slides were stained with Instant Prov (Newprov, Pinhais, Brazil) and attached to glass slides using Entellan (Merck, Darmstadt, Germany), and photomicrographs (400 ×) were captured using light microscopy (Carl Zeiss, Oberkochen, Germany).

### Infection of IFN-γKO mice with *M*. *abscessus* subsp. *massiliense*

The animals were intraperitoneously anaesthetized with a mixture of 70mg/kg of ketamine and 7mg/kg of xylazine followed by intravenous infection in the retro-orbital plexus with 10^6^ CFU of *M*. *abscessus* subsp. *massiliense* GO 06. The bacteria load in the mice organs was monitored at days 1 and 18 post infection. At day one post infection, one animal from each treatment group (total of 6 animals) was euthanized to determine the efficiency of the infection. The animals were routinely (twice daily) inspected for mortality during 18 days. Animal health was monitored for the following signs: loss of appetite, dehydration, prostration or lethargy. A humane endpoint protocol to perform euthanasia of animals presenting any of the above symptoms was approved the Ethical Committee on the animal use of Universidade Federal of Goiás. None of the animals presented any of the above signs or died during the experiments. Eighteen days after infection, the lungs, liver and spleen from one animal of each treatment group were homogenized in PBS containing 0.05% of Tween 80, and the bacterial load of the organs was determined as previously described [19]. Briefly, the lungs, liver and spleen were homogenized in 5 mL of PBS with 0.05% of Tween 80. The organ homogenates were serially diluted in PBS/Tween 80, plated onto MH agar plates and incubated for 7 days at 35°C. The colonies grown on MH agar plates were counted, and the bacterial load in each organ was determined after correcting the values based on dilution, plating volume, and the volume of homogenates.

### Treatment of IFN-γKO mice infected with *M*. *abscessus* subsp. *massiliense*

The Polydim-I treatment scheme for *M*. *abscessus* subsp. *massiliense* was performed according to [21] with some modifications. Briefly, anesthetized IFN-γKO mice were intravenously infected with *M*. *abscessus* subsp. *massiliense* (10^6^ CFU/mL). Prior to treatment, the animals were individually weighed, and the mean live weight (mLW) was used to calculate the treatment dose. After 18 days of infection, the animals (4 animals/group) were treated once a day intraperitoneally for eight days. During the treatment, the animals were monitored for signs of sickness as state above. For the Polydim-I treatment, three groups received the following dose treatments: 2 mg/kg/mLW, 1 mg/kg/mLW, and 0.5 mg/kg/mLW [2, 22]. As controls, a group was treated with CLR (200 mg/kg/mLW, treatment control) and another group received PBS (infection control). None of the animals became clinically ill or died during the experiments. To minimize pain and distress the animals were quickly handled by trained researchers. All animals were humanely euthanized by cervical dislocation by a veterinarian, one day after the end of the treatments, and the spleen, lungs and liver were collected separately and frozen for CFU determination. The experiments were repeated three times.

### Histopathological analysis

Twenty six days after intravenous infection, the right caudal lung lobes were removed and fixed in 10% buffered formaldehyde. To evaluate *in vivo* acute cytotoxicity effect of Polydim I, BALB/c mice treated with 2 or 20 mg/kg/mLW and the livers were analyzed 48 hours later. Five micrometers thick slices were stained with Hematoxilin and Eosin (H.E.) and analyzed with light microscopy (Leica DM/LS IV 500 microscopy system), and images were processed with LAS software version 4.4 (Leica). A certified Pathologist blinded to original section sources evaluated the slides. Scores were determined based on the area with lesions relative to the area of the total visual field using AxioVision microscopy Software. The results are presented as the percentage of area with lesions. Three different fields were evaluated per slide for each animal from each group.

### Statistical analysis

The experimental data were tabulated and grouped using the Microsoft Excel 2010 software and exported to the Graph Pad Prism 5.0 software (Graph Pad Software, San Diego CA, USA) for statistical analysis. The results were compared using one-way ANOVA, followed by Dunn’s test. p-values less than 0.05 were considered statistically significant. Data are available as online files (S1–S4 Figs).

## Results

### Separation, identification and synthesis of Polydim-I

**Fig 1:**
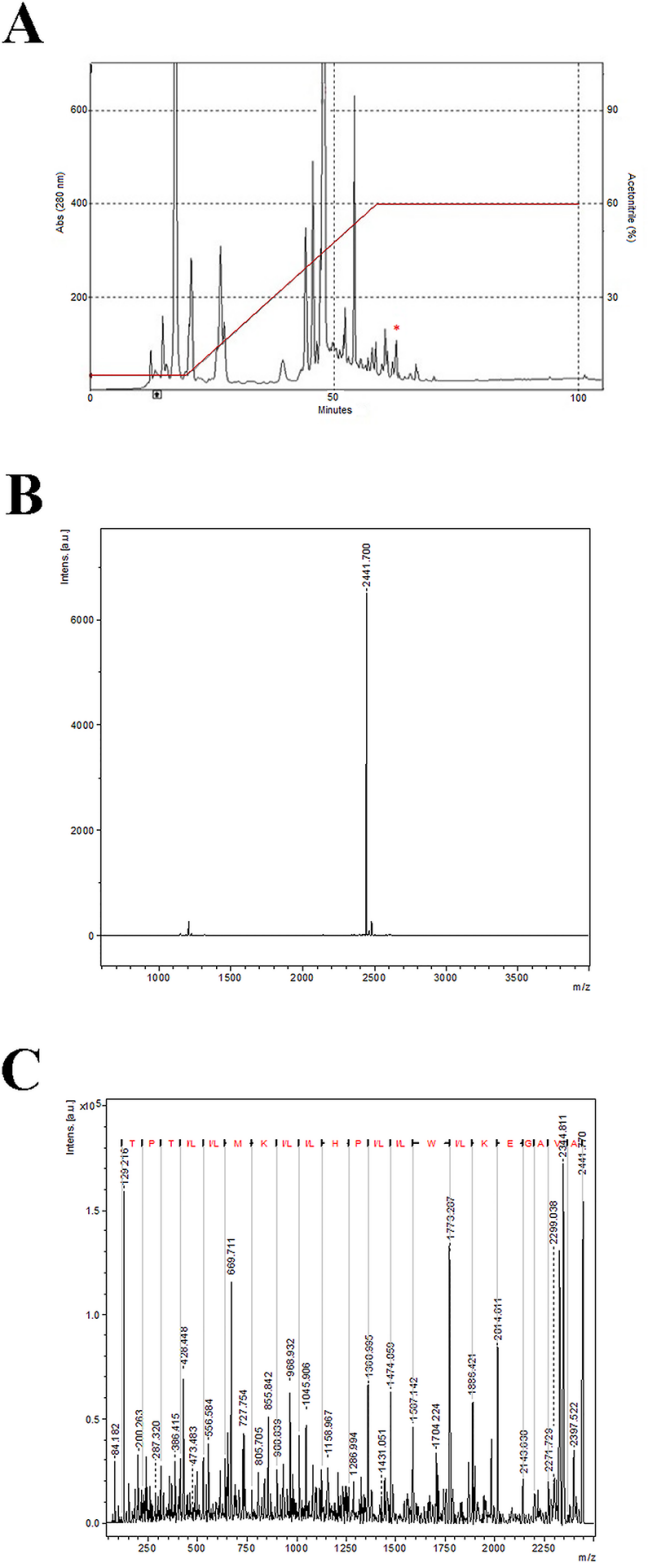
Chromatographic profile, mass spectra and *de novo* sequencing of Polydim-I peptide. (A). Chromatographic profile obtained after separation of the low molecular weight compounds from *Polybia dimorpha* venom through reversed phase high performance liquid chromatography. The fraction containing the peptide Polydim-I is signalized. (B) Mass spectra of fraction 12 obtained through MALDI TOF/TOF, with the major ion m/z 2441.7 [M+H]+. (C). *De novo* sequencing of Polydim-I using MS/MS. The ion series y is written above with the 22 amino acids represented by letters.

A total of 684 venom glands were obtained from a single nest of *Polybia dimorpha* and provided 21.4 mg of ultrafiltered crude venom (compounds with molecular masses lower than 3,000 Da). The obtained RP-HPLC profile comprised 13 fractions (Fig 1A), which were termed Poly 1–13. Two chromatographic procedures with injections of 200 μL each were performed, and both profiles showed the same fraction elution pattern. Polydim-I corresponded to fraction Poly 12, eluted at a retention time of approximately 62 min at 60% of ACN as indicated in Fig 1A.

Fraction 12 had a major molecular component at m/z 2441.7 [M+H]^+^ based on the mass spectrometry analysis, and this peptide was named Polydim-I (Fig 1B). *De novo* sequencing of the fragmented ion through MS/MS resulted in a sequence of 22 amino acid residues (AVAGEKLWLLPHLLKMLLTPTP), as shown in Fig 1C.

Thus, Polydim-I was synthesized at Aminotech Development and Technology LTDA. The purity (> 99%) and the correct sequence of the peptide were verified and confirmed through mass spectrometry upon receipt using the same parameters that were used for the natural compound.

### *In vitro* antimycobacterial and morphology modification activities of Polydim-I against *M*. *abscessus* subsp. *massiliense*

**Fig 2:**
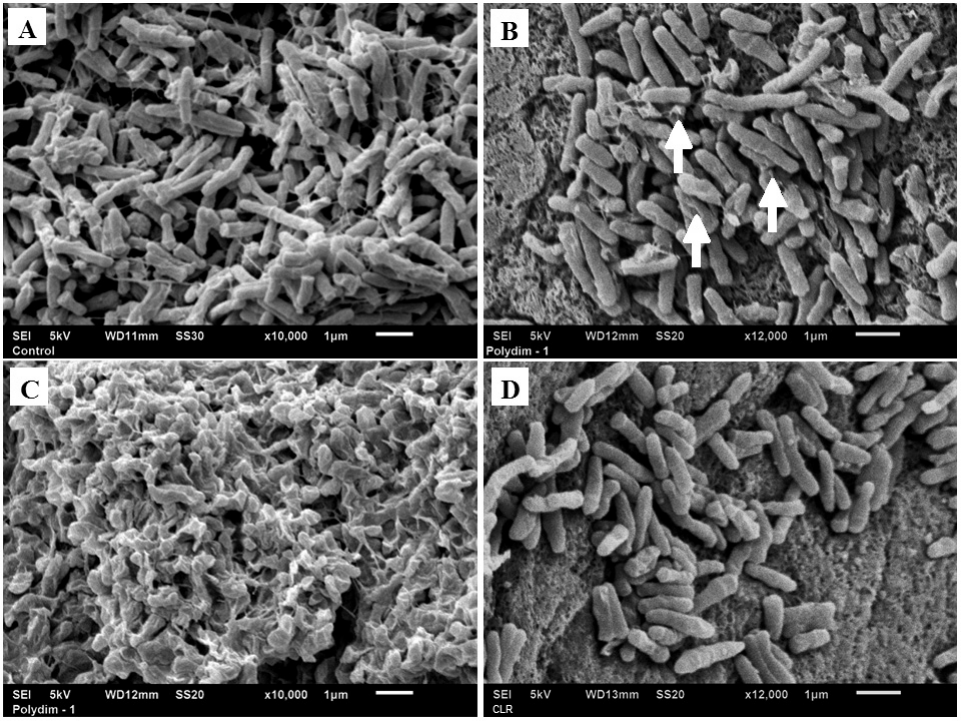
Morphology of *M*. *abscessus* subsp. *massiliense* cell surface upon the action of Polydim-I peptide. (A) Scanning Electron microscopy of untreated *M*. *abscessus* subsp. *massiliense* cells. (B) *M*. *abscessus* subsp. *massiliense* exposed to 3.8 μg/mL of Polydim-I peptide for 24 hours, presenting mild surface alterations (white arrows). *M*. *abscessus* subsp. *massiliense* with severe cell wall disruptions after treatment with 7.6 μg/mL of Polydim-I peptide for 24 hours (C). (D) *M*. *abscessus* subsp. *massiliense* treated with clarithromycin (0.5 μg/mL) without visible cell wall damage. (A) and (C) 10,000 × magnification. (B) and (D) 12,000 × magnification.

The Polydim-I antimycobacterial activity was evaluated against six isolates of *M*. *abscessus* subsp. *massiliense* from an epidemic outbreak and two reference strains resulting in a MIC of 60.8 μg/mL, while treatment at a concentration of 15.2 μg/mL inhibited 55 to 68% of *M*. *abscessus* subsp. *massiliense* strains growth (data not shown). To determine whether the mycobactericidal effect of Polydim-I could influence the shape of the cell envelope, scanning electron microscopy (SEM) of *M*. *abscessus* subsp. *massiliense* GO 06 exposed to the peptide was performed. As shown in Fig 2, the cellular shape and cell wall integrity were slightly affected when the mycobacteria were exposed to 7.6 μg/mL of the peptide (Fig 2B, arrow heads). The cell shape was expressively damaged when the concentration of the peptide was 15.2 μg/mL (a representative damaged area is shown in Fig 2C). The exposure of *M*. *abscessus* subsp. *massiliense* to the antibiotic CLR, which prevents bacterial growth through the inhibition of protein synthesis, did not result in visible morphological changes (Fig 2D). Taken together, the SEM data suggest that Polydim-I might elicit deleterious effects through perturbations of the cell integrity.

### Polydim-I displays bactericidal activity on intracellular environment

**Fig 3:**
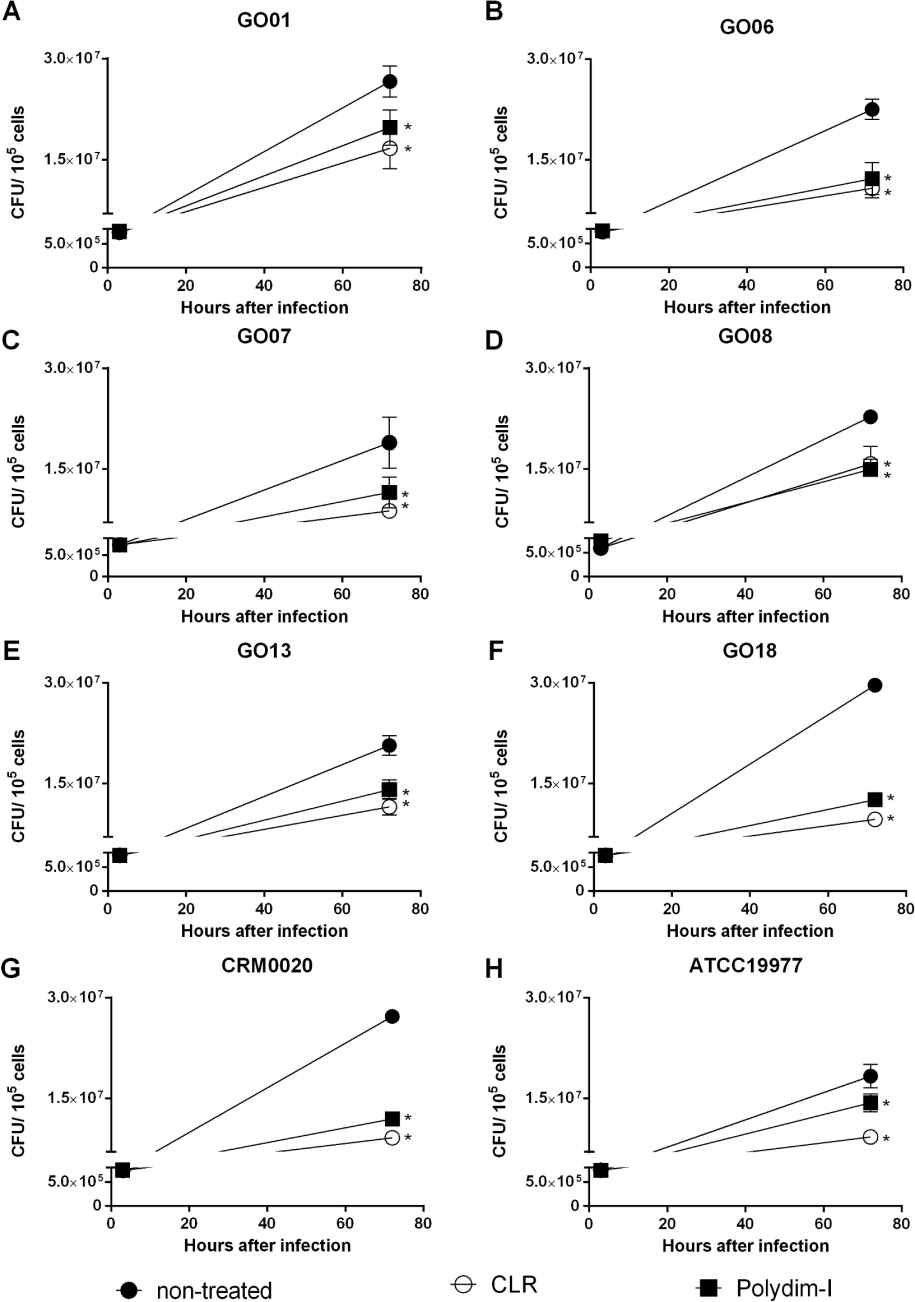
*M*. *abscessus* subsp. *massiliense* CFU in infected macrophages treated with Polydim-I peptide. Peritoneal macrophages from BALB/c mice were infected with 6 isolates of *M*. *abscessus* subsp. *massiliense* (A thru F) or the reference strains CRM0020 (G) and ATCC19977 (H) at a MOI of 10:1, and treated with 7.6 μg/mL of Polydim-I peptide or with 1 μg/mL of clarithromycin. Bacterial load of phagocytosed bacteria were determined at three hours after infection and total recovered bacteria from supernatant and macrophages at 72 hours after infection were determined for non-treated (closed circles), peptide treated (closed squares), and clarithromycin treated (open circles). The data represent mean of quadruplicates and is representative of three independent experiments. One-way ANOVA followed by Dunn’s test was used to determine significant differences (* p<0.05).

**Fig 4:**
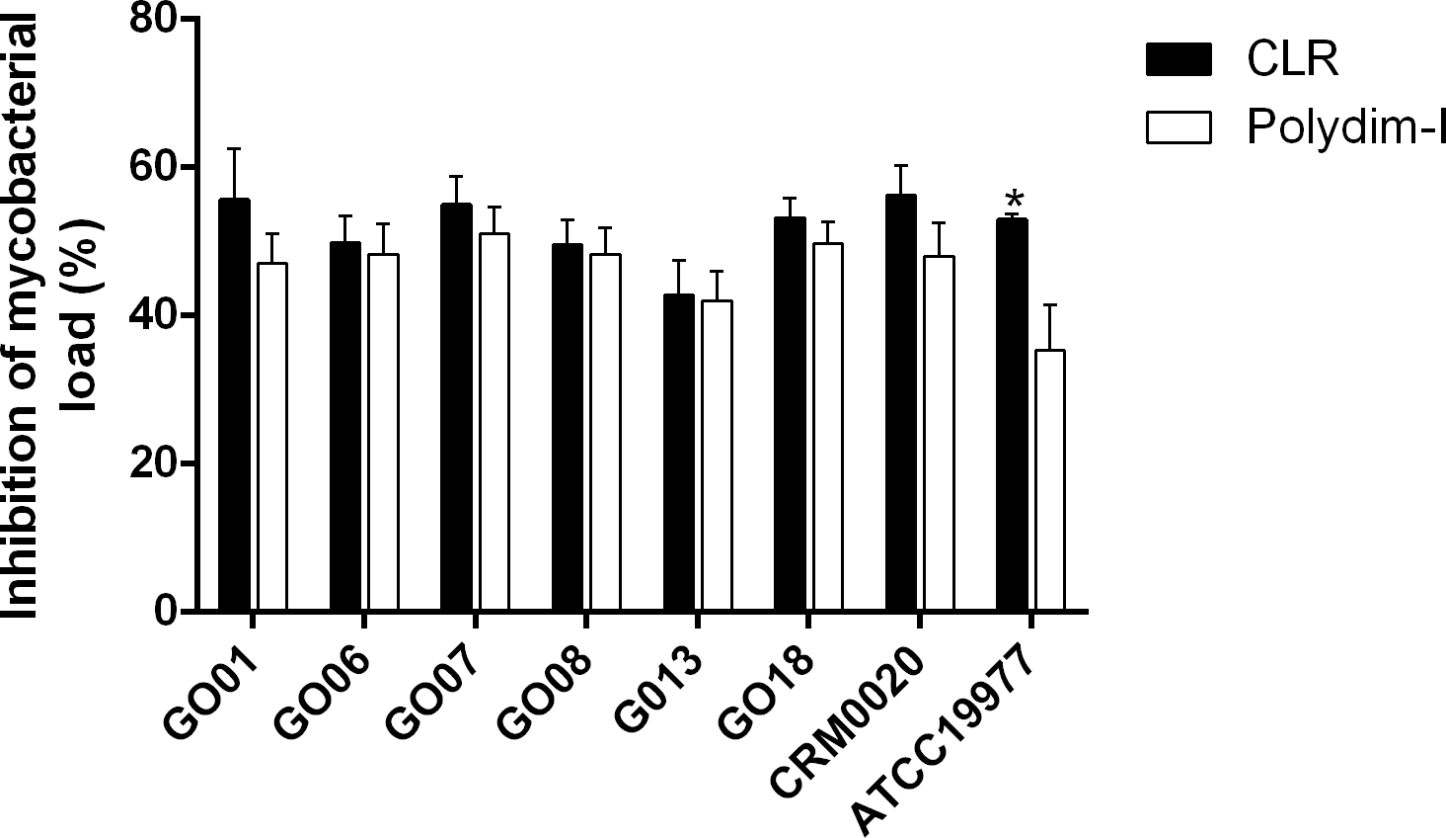
Percentage of mycobacterial load reduction in infected macrophages by Polydim-I treatment. The CFU in peritoneal macrophages, 72 hours after treatment with Polydim-I (white bars) or clarithromycin (black bars), was determined and the percentage of inhibition was calculated in relation to non-treated macrophage cultures. The data represent mean of quadruplicates and is representative of three independent experiments. One-way ANOVA followed by Dunn’s test was used to determine significant differences (* p<0.05).

To further investigate whether a combination of mycobacteria infection and peptide treatment could affect the membrane integrity of peritoneal macrophages, these cells were infected with six different *M*. *abscessus* subsp. *massiliense* isolates or the reference strains CRM0020 and ATCC19977 (at MOI 10:1) and treated with 7.6 μg/mL of Polydim-I. All tested strains behaved similarly, and as shown in S5A Fig (control) and S5B (treated), the peptide treatment of peritoneal macrophages apparently did not affect the cell morphology, which was not altered after infection with the representative *M*. *abscessus* subsp. *massiliense* isolate GO 06 shown in S5C and S5D Fig. The macrophage morphology suggests that they were viable throughout the experiments. Additionally, CFU counting of infected and Polydim-I treated macrophages showed a significant reduction in the bacterial load when compared to the infected non-treated macrophages (40 to 50% range; Figs 3 and 4). Infected macrophages treated with 1μg/ml of CLR reduced 57–70% the bacterial load among the different tested strains (Fig 4).

**Fig 5:**
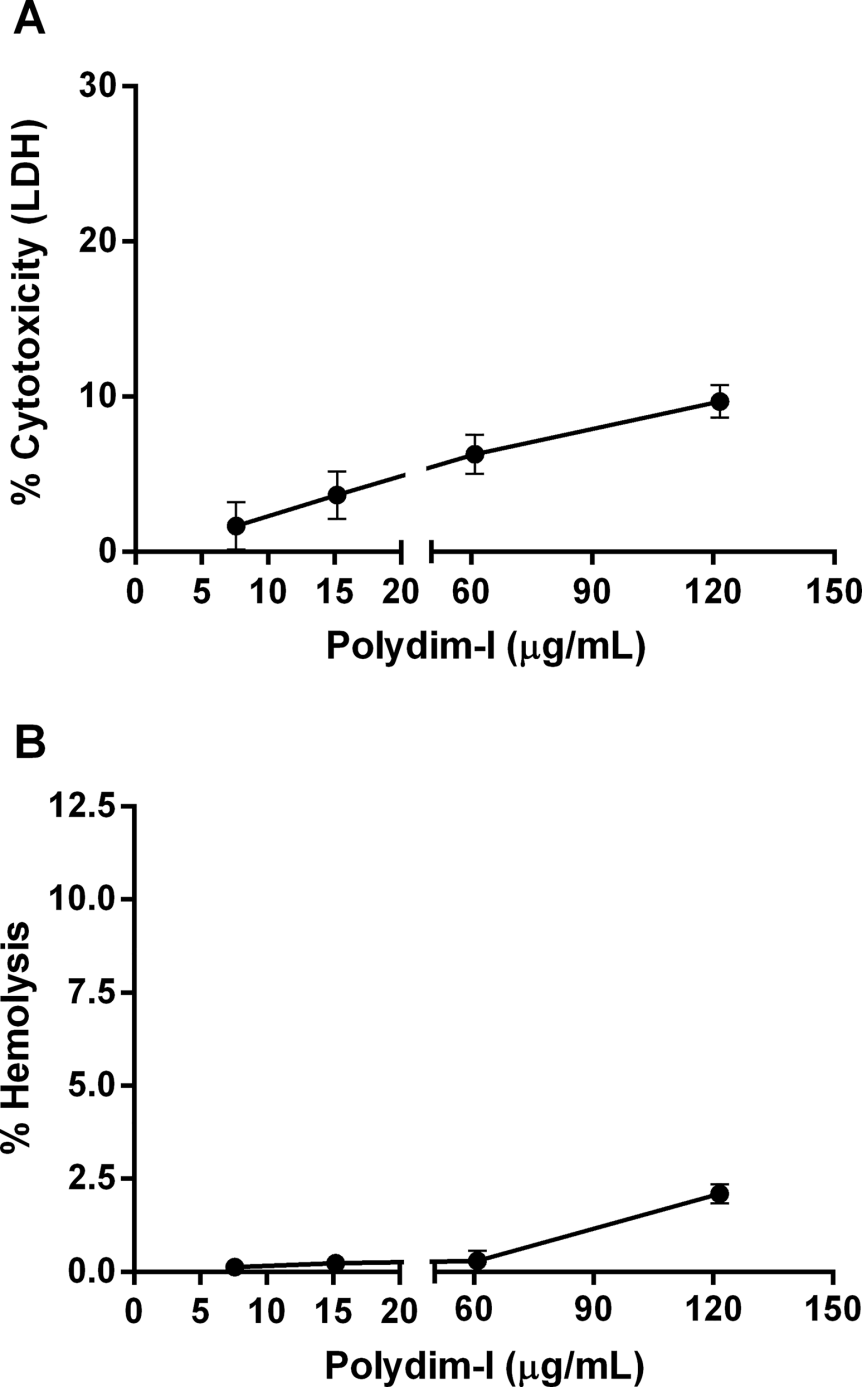
J774 macrophage cell line cytotoxic effects and hemolytic activity of Polydim-I peptide. J774 macrophages were exposed to different concentrations of Polydim-I peptide (7.6, 15.2, 60.8, or 121.6 μg/mL) for 3 days. Cell viability percentage was determined by LDH release assay (A). In (B), the percentage of human red blood cells lysis. The results represent three independent experiments.

As reasoned above, Polydim-I activity might be associated with the disruption of cell membrane integrity. Nonetheless, to be a potential candidate as an antimicrobial agent, this compound must show selective toxicity (12). Consequently, we analyzed whether Polydim-I interferes with eukaryotic cell membranes using J774 macrophage cell line treated with 7.6, 15.2, 60.8, and 121.6 μg/mL of Polydim-I for 24 hours using LDH release assay. Polydim-I started to present cytotoxicity on J774 cells at concentrations above 121.6 μg/mL (10%) (Fig 5A). However the Polydim-I peptide at concentrations up to 121.6 μg/mL presented less than 2.5% of hemolytic activity (Fig 5B).

### *In vivo* anti-mycobacterial activity of Polydim-I

**Fig 6:**
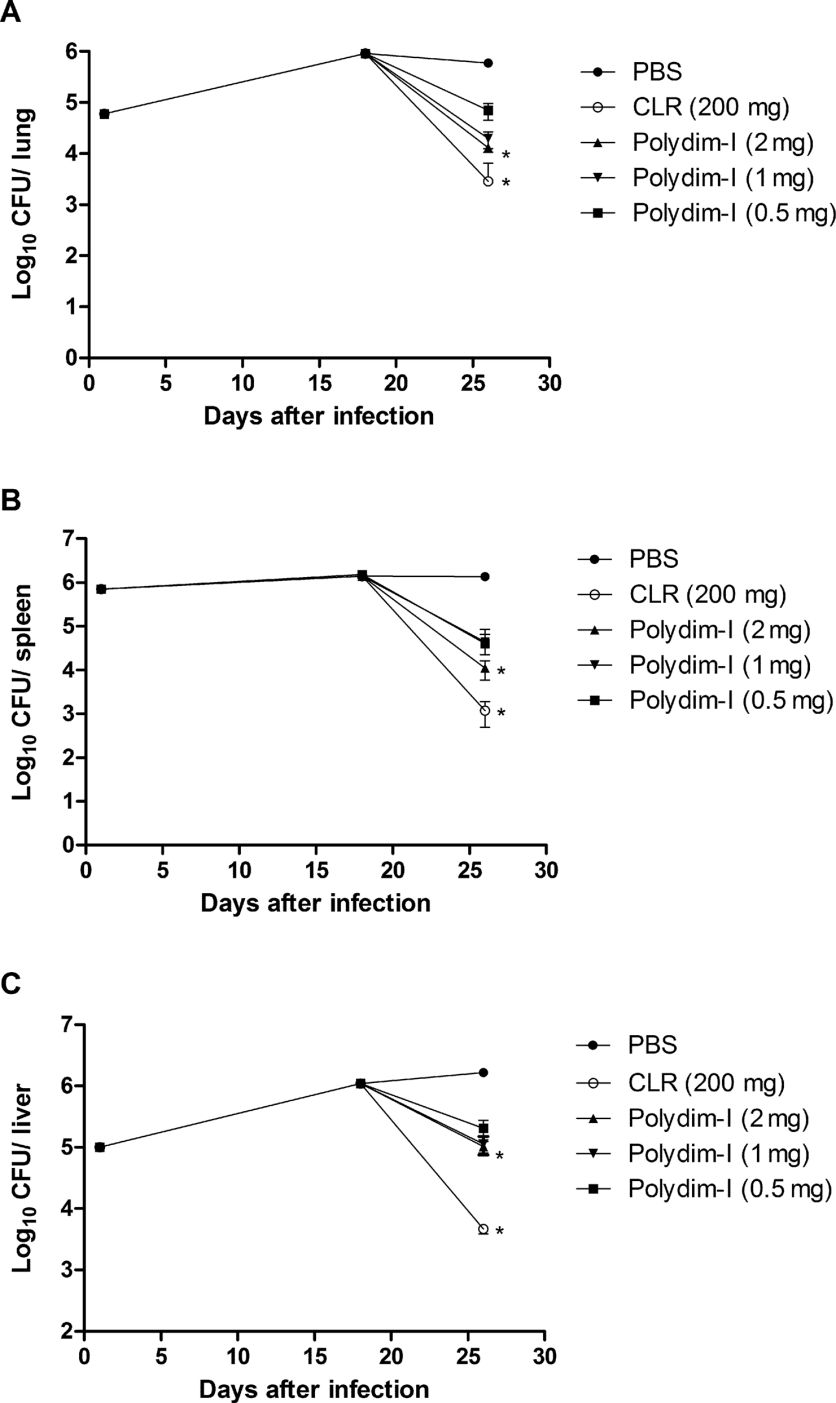
*M*. *abscessus* subsp. *massiliense* bacillary load reduction in IFN-γKO-infected mice after treatment with Polydim-I peptide. IFN-γKO mice were infected intravenously with 10^6^ CFU of *M*. *abscessus* subsp. *massiliense* strain GO 06. Eighteen days after infection, the mice were treated with 2 mg/kg/mLW of Polydim-I (▲), 1 mg/kg/mLW of Polydim-I (▼), 0.5 mg/kg/mLW (■) of Polydim-I, or with 200 mg/kg/mLW of clarithromycin (CLR, ○). As control, a group of infected mice were treated with PBS (●). The bacillary loads in the lungs (A), spleen (B) and liver (C) were determined after euthanizing the mice at days 1, 18 and 26 post infection. The data represent mean of four mice per group and is representative of three independent experiments. One-way ANOVA followed by Dunn’s test was used to determine significant differences. Statistically significant difference when compared to PBS treated control group (* p<0.05).

**Fig 7:**
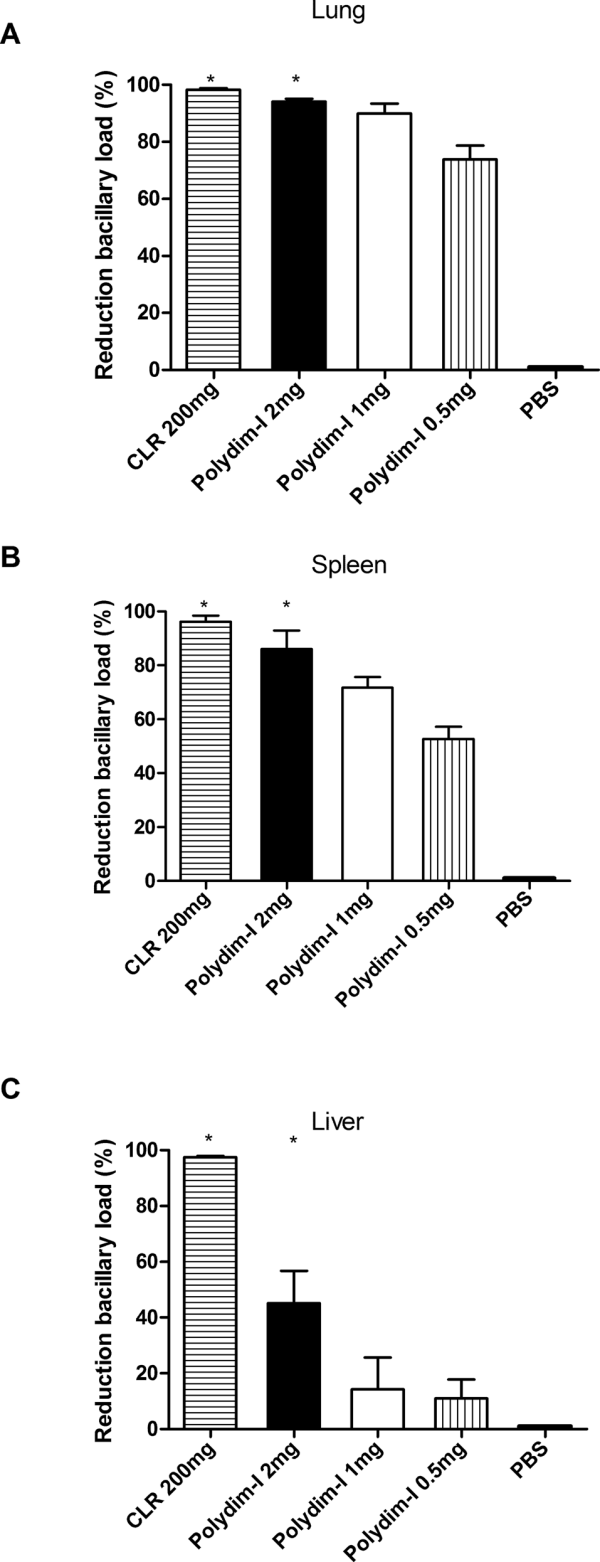
*M*. *abscessus subsp*. *massiliense* bacillary load reduction in IFN-γKO-infected mice after treatment with Polydim-I peptide. IFN-γKO mice were infected intravenously with 10^6^ CFU of *M*. *abscessus* subsp. *massiliense*. Eighteen days after infection, the mice were treated with Polydim-I peptide at three different dosages (2, 1 or 0.5 mg/kg/mLW) or with clarithromycin (CLR 200 mg/kg/mLW). As a control group, infected mice were treated with PBS. Percentages of bacterial load reduction in the lungs (A), spleen (B) and liver (C) from each treatment group were determined relative to the control PBS group (* p<0.05).

**Fig 8:**
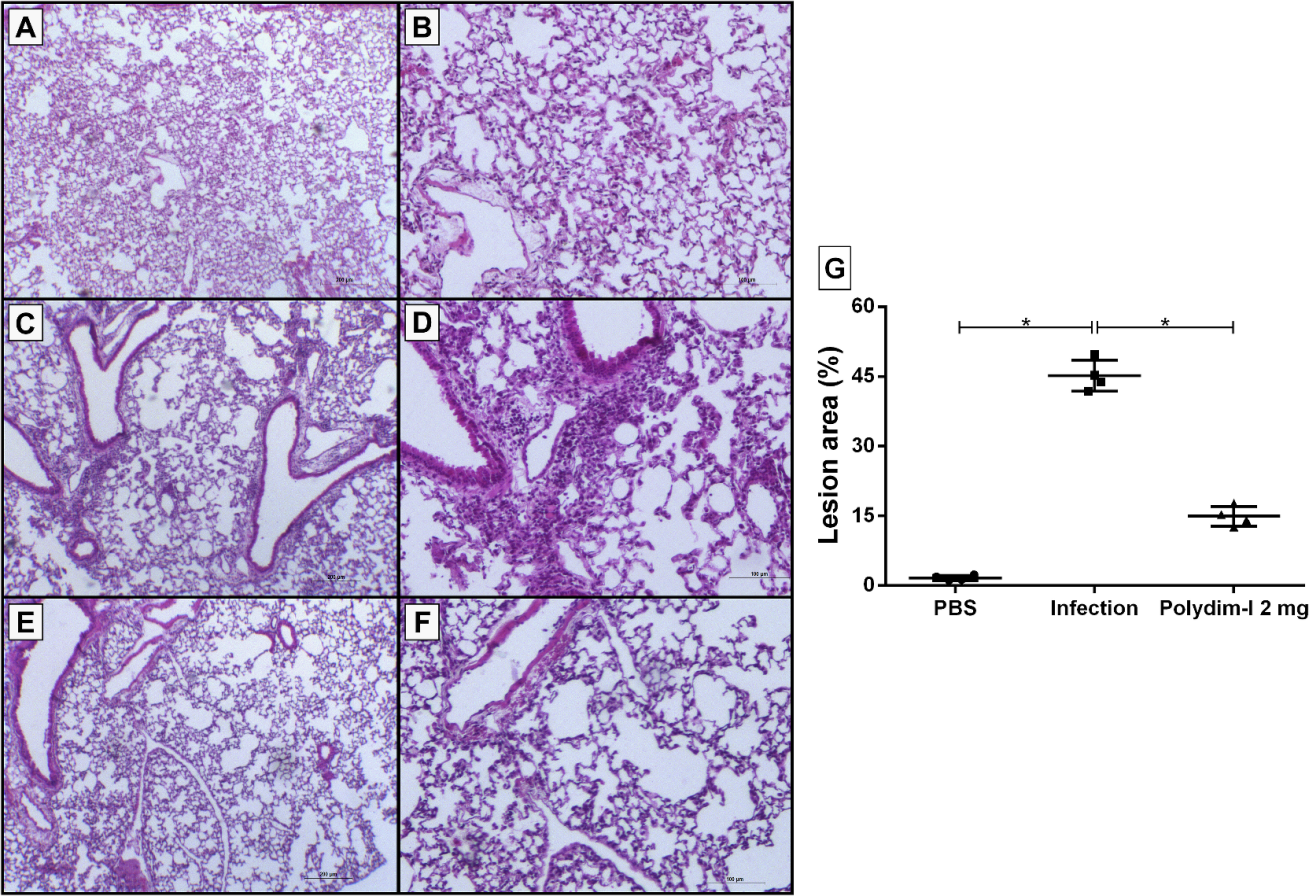
Lung inflammatory lesions caused by *M*. *abscessus* subsp. *massiliense* infection were reduced by Polydim-I peptide treatment. Twenty-six days after the challenge, the lungs were processed, sectioned, HE stained, and examined at 40 × or 100 × magnification. (A and B) normal lungs from PBS treated control group at 40 × and 100 × magnifications, respectively; inflammatory lesions observed in the lungs of the infected group 40 × (C) and 100 × (D). Lungs from animals infected and treated with Polydim-I (2 mg/kg/mLW) 40 × (E) and 100 × (F). The area of the inflammatory lesions were calculated and plotted in a graph (G). One-way ANOVA followed by Dunn’s test was used to determine significant differences (* p<0.05).

We have previously shown that BALB/c or C57BL/6 intravenous infection with *M*. *abscessus* subsp. *massiliense* strain GO 06 (10^6^ CFU) provided an adequate model of infection with the bacterial load maintained until 30 days post infection [19]. To optimize the analysis of the peptide treatment effect *in vivo*, IFN-γKO mice, that have increased susceptibility to mycobacterial infection, were infected with *M*. *abscessus* subsp. *massiliense*. Eighteen days post infection, these animals were treated for 8 days with CLR (200 mg/kg/mLW) or one of three different doses of Polydim-I (2, 1 or 0.5 mg/kg/mLW) for 8 days. Fig 6 shows the CFU obtained after 28 days post infection with *M*. *abscessus* subsp. *massiliense* and the different treatment schemes. As shown, Polydim-I treatment at 2 mg/kg/mLW showed significant reduction of the bacterial load in all analyzed organs. The peptide treatment at this concentration reduced the bacillary load of the susceptible animals by up to 90% in the lungs and spleen (Fig 7). In addition, Polydim-I treatment of mice infected with *M*. *abscessus* subsp. *massiliense* reduced the inflammatory response caused by the infection as determined by the quantification of the area occupied by lesion/inflammatory responses (Fig 8). The highest dose of Polydim-I used to treat infected mice did not show damages associated with cytotoxicity in the lungs or liver (Data not shown and S6 Fig).

## Discussion

*M*. *abscessus* subsp. *massiliense* presents resistance to several of the conventional treatment regimens that are typically used to treat *M*. *tuberculosis*. Therefore, the identification of new drugs to improve the current treatment or lower the risk of inducing resistance is urgently needed. In this context, the present work shows the first use of a peptide derived from wasp venom that possesses mycobactericidal activity. Polydim-I provoked cell wall disruption and presented low cytotoxicity towards mammalian cells. The treatment of *M*. *abscessus* subsp. *massiliense*-infected macrophages with the peptide reduced approximately 50% of the bacterial load. Additionally, Polydim-I treatment of highly susceptible mice infected with *M*. *abscessus* subsp. *massiliense* induced a reduction of the bacterial load in the lungs, spleen, and livers.

Polydim-I, which was a peptide, isolated from the venom of *Polybia dimorpha*, presents characteristics similar to other wasp AMPs (Fig 1), with a structure comprising a series of hydrophobic amino acid residues, such as methionine, leucine, valine, and proline, which are intercalated with negatively charged residues to create an amphipathic surface [23, 24]. However, due to the intrinsic characteristics observed in the sequence of this peptide, it cannot be classified as mastoparan and has subsequently emerged as a new class of peptides within the major group of AMPs. Some peptides isolated from other wasps, such as MP-AF and MPI, exhibit interesting antimicrobial activity against Gram-positive and Gram-negative bacteria [25] as well as antifungal activity against *Candida albicans* and *Candida glabrata* yeasts [26]. Natural peptides, such as RR-11, a cationic host defensin, have been demonstrated to have activity against *M*. *smegmatis* [27] when used at a concentration of 125 μg/mL. Our findings are promising, as using a much lower concentration of peptide (7.6 μg/mL) achieved a 50% reduction of mycobacterial growth. Although we have included in our experiments Tween 80 to avoid clumping, a factor that has been shown to disrupt mycobacteria cell wall integrity before [28], we believe that that inhibitory effect is mainly due to the peptide because all controls also were grown with the detergent.

The treatment of *M*. *abscessus* subsp. *massiliense* with Polydim-I disrupted the bacteria cell envelope (Fig 2); a similar phenomenon was observed when *Candida* sp. [26] were treated with the wasp MPI peptide, which caused cell wall disruptions. Defensin RR-11 also promoted *M*. *smegmatis* cell wall destruction [29]. LL-37, a cathelicidin that is secreted from macrophages, exhibited anti-mycobacterial activity, further strengthening the potential use of these agents against mycobacteria [30]. In a recent review, Padhi and colleagues suggested that the mechanism of action of natural peptides showing activity against mycobacteria involves the destruction of cell envelope structures and/or functions through associations with lipoarabinomannan [30, 31]. Additionally, bacterial membranes have unique lipids such as phosphatidylglycerol, cardiolipin or phosphatidelserine conferring a negative charge that may favor AMP association [25]. Thus, the activity of Polydim-I is likely associated with cell envelope or cell wall damage because this effect was primarily observed with other microorganisms [25, 26], but further studies in this field are needed.

Macrophages are the main targets of mycobacteria and are morphologically and functionally well characterized; hence, these cells were used in cytotoxicity assays (J774 macrophages) and to evaluate *in vitro* Polydim-I action against *M*. *abscessus* subsp. *massiliense* phagocytosed by macrophages. The membrane and cell envelope interference properties of natural peptides could affect eukaryotic cells [32], but the peptide used in the present study did not exhibit any expressive interaction or destruction of eukaryotic cell membranes at concentrations ≤60.8 μg/mL. Natural peptides such as cathelicidin also presented selective toxicity to mycobacteria without damaging macrophage membranes [33], and this might be associated with the cholesterol and other lipid contents of eukaryotic membranes [31]. In addition, cationic AMPs interact with negatively charged membranes, such as seen among bacteria and tumoral cells [34, 35] forming pores, a role most likely performed by the arginine amino acid residues present in the peptides [34]. In contrast, the membranes of normal eukaryotic cells have a predominant neutral charge and consequently could be resistant to the AMPs action. The results of our research describes for the first time, a peptide isolated from the venom of wasps with activity against mycobacteria and although it presented some *in vitro* cytotoxicity, it can be further modified to improve its efficacy (reduce cytotoxicity and increase mycobactericidal activity). The *in vivo* selective action presented by Polydim-I could be due to the presence of two arginine residues present in its N-terminus. These results are promising because for other wasp AMPs, particularly mastoparans such as MP-AF, MP-A, MP-B, MP-D, MP-M, and MP-V, high cell toxicity through hemolysis was observed [24]; however, the results obtained in the present study showed a low level of hemolysis.

Treatment of infected macrophages with 7.6 μg/mL of Polydim-I decreased approximately 50% of the bacterial load. Although few studies have used infected macrophages to test new drugs against mycobacteria [36], no scientific publication, to our knowledge, has examined new drugs using macrophages that were infected with *M*. *abscessus* subsp. *massiliensi*, previously known as *M*. *massiliense* [4]. We hypothesize that an efficient drug will need to penetrate granuloma and should not be eliminated through efflux pumps [37] in order to eliminate the mycobacteria within the infected macrophages [36]. Therefore, infected macrophages are also an effective model to evaluate the mechanism of action of antimycobacterial drugs.

Because this is the first study evaluating the treatment of mice infected with *M*. *abscessus* subsp. *massiliense*, we used the mouse model of Lenaerts and colleagues [21] to test drugs against *M*. *tuberculosis*. IFN-γKO mice are highly susceptible to mycobacteria infections [38], particularly infections with *M*. *abscessus* subsp. *massiliense* [7], which facilitates a more rapid treatment result. Additionally, we used parenteral drug treatments in order to guarantee homogeneous bioavailability for all drugs as proposed before [22, 23]. The treatment of IFN-γKO mice infected with *M*. *abscessus* subsp. *massiliense* using 2 mg/kg/mLW of Polydim-I substantially reduced the bacterial load in the lungs, spleen and livers (Figs 6 and 7). Importantly, when clarithromycin was used, the *M*. *abscessus* subsp. *massiliense* was not cleared from IFN-γKO-infected mice; this finding indicates that, as observed with human RGM infection, a multidrug therapy is always needed to avoid bacterial persistence and possibly drug resistance development [39, 40]. The results obtained in the present study are consistent with those obtained for several drugs that have been demonstrated as useful for treating mycobacterial infections [36, 41]. It will be interesting to evaluate the effect of Polydim-I treatment combined with other drugs.

Lungs from animals infected with *M*. *abscessus* subsp. *massiliense* and treated with Polydim-I showed less inflammatory lesions than the lungs from infected animals. Although, no work have used IFNγ-KO to test new drugs to treat *M*. *abscessus* subsp. *massiliense* infection, others have shown that depending on the peptide used, the bacterial load reduction is accompanied or not by reduction of the inflammatory lesions induced by *M*. *tuberculosis* infection [29, 42]. It is interesting to note that the treatment with Polydim-I using doses ranging from 10 to 40 μg per animal, doses bellow the MIC, were sufficient to reduce approximately one Log of the bacterial burden. Other authors have shown that AMP activate macrophages enhancing their bactericidal activity as well as cytokine production [14, 43, 44]. Here we hypothesize that Polydim-I might activate the infected macrophages potentiating the mycobacteria clearance.

*M*. *abscessus* subsp. *massiliense* is susceptible to parenteral drugs (amikacin, cefoxitin, and imipenem) and oral macrolides (clarithromycin and azithromycin). Consequently, the ATS/IDSA (American Thoracic Society/Infectious Disease Society of America) recommends that at least two parental drugs and one oral drug should be used to treat infections to avoid drug resistance development [20]. These drugs cause several collateral effects, such as gastric intestinal dysfunction, ototoxicity, and neurotoxicity [45], and the use of clarithromycin has been associated with a significantly increased risk of cardiac death [46]. Novel drugs, which target different structures compared with most of the commercially available drugs, could be incorporated into multi-regimen treatments, thereby reducing the dosage and/or number of drugs used and consequently lowering the risk of developing side effects. Therefore, Polydim-I could improve the treatment of *M*. *abscessus* subsp. *massiliense* infections because of the lower risk of resistance development and the lower cost of production.

Some important aspects about the use of AMPs in general should be pointed out as for example they could be degraded faster than other anti-microbial agents due to the action of tissue peptidases/proteinases *in vivo*. Additionally, peptides could combine to host proteins and generate specific humoral immune responses that could impair repetitive use of the peptide [47–49].

In conclusion, this study was the first to demonstrate the therapeutic potential of a peptide derived from wasp venom to treat mycobacteria infections. Polydim-I acts on the *M*. *abscessus* subsp. *massiliense* cell wall and reduced the bacterial load both *in vitro* and *in vivo*. These results encourage further studies on the use of Polydim-I and its derivative peptides as a component in the *M*. *abscessus* subsp. *massiliense* infection treatment formulation.

## Supporting Information

S1 Fig MIC determination raw data experiment. (PDF)

S2 Fig Raw data from *M*. *abscessus subsp*. *massiliense* CFU in infected macrophages treated with Polydim-I peptide experiment. (PDF)

S3 Fig Raw data from J774 macrophage cell line cytotoxic effects and hemolytic activity of Polydim-I peptide experiment. (PDF)

S4 Fig Raw data from *M*. *abscessus subsp*. *massiliense* bacillary load reduction in IFN-γKO-infected mice after treatment with Polydim-I peptide experiment. (PDF)

S5 Fig Macrophage morphology after Polydim-I treatment and infection. Instant-Prov staining of: (A) Untreated macrophages; (B) Polydim-I peptide-treated macrophages; (C) macrophages infected with *Mycobacterium abscessus* subsp. *massiliense* isolate GO 06 (MOI 10:1); and (D) macrophages infected with *M*. *abscessus* subsp. *massiliense* isolate GO 06 and treated with Polydim-I peptide.
(TIF)

S6 Fig Histological analyses of the liver from mice treated with Polydim-I. BALB/c mice were treated with 2 mg/kg/mLW (C and D) or 20 mg/kg/mLW (E and F) and 48 hours later the livers were processed and stained with HE. As controls, mice were treated with PBS (A and B). The results are presented at 40 x and 100 x magnification.
(TIF)
