# Supplementary material for: Antimycobacterial Activity of a New Peptide Polydim-I Isolated from Neotropical Social Wasp Polybia dimorpha
Source: PLoS One. 2016 Mar 1;11(3):e0149729. doi: 10.1371/journal.pone.0149729 (PMC4773228; doi:10.1371/journal.pone.0149729)
Supplement: S1 Fig — (PDF) [file pone.0149729.s001.pdf]

## Experiment RCN MIC01 - June 15

**Plate 1.1**

Number of CFU / mL x 10ˆ4 per well

| Polydim Dilution (µg/mL) | | GO 01 | GO 01 | GO 06 | GO06 | GO 07 | GO 07 | GO 08 | GO 08 | ATCC19977 | CRM0020 | | GO 06 Clarithromycin | Dilution (µg/mL) |
|---|---|---|---|---|---|---|---|---|---|---|---|---|---|---|
| | | 1 | 2 | 3 | 4 | 5 | 6 | 7 | 8 | 9 | 10 | 11 | 12 | |
| | A | | | | | | | | | | | | | |
| 243.2 | B | 0 | 0 | 0 | 0 | 0 | 0 | 0 | 0 | 0 | 0 | | 0 | 8 |
| 121.6 | C | 0 | 0 | 0 | 0 | 0 | 0 | 0 | 0 | 0 | 0 | | 0 | 4 |
| 60.8 | D | 0 | 0 | 0 | 0 | 0 | 0 | 0 | 0 | 0 | 0 | | 0 | 2 |
| 30.4 | E | 2.24 | 2.64 | 6.16 | 4.08 | 0.88 | 0.56 | 1.68 | 1.76 | 0.8 | 1.52 | | 0 | 1 |
| 15.2 | F | 8 | 8.24 | 12.4 | 13.92 | 3.36 | 2.4 | 5.36 | 5.44 | 4.64 | 4.4 | | 1.04 | 0.5 |
| 7.6 | G | NC | NC | NC | NC | NC | NC | NC | NC | NC | NC | | 3.6 | 0.25 |
| 3.8 | H | NC | NC | NC | NC | NC | NC | NC | NC | NC | NC | | 7.04 | 0.125 |

Number of CFU / mL x 10ˆ4 per well
NC Not counted
0 = No growth
MIC

**Plate 1.2**

| Polydim Dilution (µg/mL) | | GO 013 | GO 13 | GO 18 | GO018 | | | ATCC19977 | CRM0020 | | | | GO 06 Clarithromycin | Dilution (µg/mL) |
|---|---|---|---|---|---|---|---|---|---|---|---|---|---|---|
| | | 1 | 2 | 3 | 4 | 5 | 6 | 7 | 8 | 9 | 10 | 11 | 12 | |
| | A | | | | | | | | | | | | | |
| 243.2 | B | 0 | 0 | 0 | 0 | | | 0 | 0 | | | | 0 | 8 |
| 121.6 | C | 0 | 0 | 0 | 0 | | | 0 | 0 | | | | 0 | 4 |
| 60.8 | D | 0.08 | 0 | 0 | 0 | | | 0 | 0 | | | | 0 | 2 |
| 30.4 | E | 3.04 | 2.64 | 4.4 | 3.68 | | | 1.2 | 2.32 | | | | 0 | 1 |
| 15.2 | F | 9.04 | 7.92 | 9.84 | 13.28 | | | 5.44 | 5.52 | | | | 1.2 | 0.5 |
| 7.6 | G | NC | NC | NC | NC | | | NC | NC | | | | 6.08 | 0.25 |
| 3.8 | H | NC | NC | NC | NC | | | NC | NC | | | | 7.12 | 0.125 |

Number of CFU / mL x 10ˆ4 per well
NC Not counted
0 = No growth
MIC

## Experiment RCN MIC02 - June 22

**Plate 2.1**

| Polydim Dilution (µg/mL) | | GO 01 | GO 01 | GO 06 | GO06 | GO 07 | GO 07 | GO 08 | GO 08 | ATCC19977 | CRM0020 | | GO 06 Clarithromycin | Dilution (µg/mL) |
|---|---|---|---|---|---|---|---|---|---|---|---|---|---|---|
| | | 1 | 2 | 3 | 4 | 5 | 6 | 7 | 8 | 9 | 10 | 11 | 12 | |
| | A | | | | | | | | | | | | | |
| 243.2 | B | 0 | 0 | 0 | 0 | 0 | 0 | 0 | 0 | 0 | 0 | | 0 | 8 |
| 121.6 | C | 0 | 0 | 0 | 0 | 0 | 0 | 0 | 0 | 0 | 0 | | 0 | 4 |
| 60.8 | D | 0 | 0 | 0 | 0 | 0 | 0 | 0 | 0 | 0 | 0 | | 0 | 2 |
| 30.4 | E | 4.64 | 7.04 | 3.04 | 4.4 | 2 | 2.32 | 5.44 | 2 | 1.52 | 1.76 | | 0 | 1 |
| 15.2 | F | 13.6 | 10.64 | 7.84 | 10.24 | 6.24 | 6.4 | 5.44 | 6.16 | 4.56 | 4.96 | | 1.84 | 0.5 |
| 7.6 | G | NC | NC | NC | NC | NC | NC | NC | NC | NC | NC | | 4.16 | 0.25 |
| 3.8 | H | NC | NC | NC | NC | NC | NC | NC | NC | NC | NC | | 7.12 | 0.125 |

Number of CFU / mL x 10ˆ4 per well
NC Not counted
0 = No growth
MIC

**Plate 2.2**

| Polydim Dilution (µg/mL) | | GO 013 | GO 13 | GO 18 | GO018 | | | ATCC19977 | CRM0020 | | | | GO 06 Clarithromycin | Dilution (µg/mL) |
|---|---|---|---|---|---|---|---|---|---|---|---|---|---|---|
| | | 1 | 2 | 3 | 4 | 5 | 6 | 7 | 8 | 9 | 10 | 11 | 12 | |
| | A | | | | | | | | | | | | | |
| 243.2 | B | 0 | 0 | 0 | 0 | | | 0 | 0 | | | | 0 | 8 |
| 121.6 | C | 0 | 0 | 0 | 0 | | | 0 | 0 | | | | 0 | 4 |
| 60.8 | D | 0 | 0 | 0.24 | 0 | | | 0 | 0 | | | | 0 | 2 |
| 30.4 | E | 3.12 | 2.32 | 0.4 | 0.56 | | | 1.2 | 3.52 | | | | 0 | 1 |
| 15.2 | F | 6.24 | 4.8 | 4.08 | 3.92 | | | 6.16 | 9.84 | | | | 2.16 | 0.5 |
| 7.6 | G | NC | NC | NC | NC | | | NC | NC | | | | 3.68 | 0.25 |
| 3.8 | H | NC | NC | NC | NC | | | NC | NC | | | | 8.08 | 0.125 |

Number of CFU / mL x 10ˆ4 per well
NC Not counted
0 = No growth
MIC
