# Supplementary material for: Antimycobacterial Activity of a New Peptide Polydim-I Isolated from Neotropical Social Wasp Polybia dimorpha
Source: PLoS One. 2016 Mar 1;11(3):e0149729. doi: 10.1371/journal.pone.0149729 (PMC4773228; doi:10.1371/journal.pone.0149729)
Supplement: S2 Fig — (PDF) [file pone.0149729.s002.pdf]

Macrophage + Polydim-I+ *M. abscessus subsp massiliense*
DATE:

GO01

Polydim-I			CTRL			CLR	
175	17500000		246	24600000		182	18200000
220	22000000		286	28600000		171	17100000
176	17600000					123	12300000
227	22700000					191	19100000

GO06

Polydim-I			CTRL			CLR	
120	12000000		238	23800000		212	21200000
160	16000000		212	21200000		95	9500000
115	11500000					120	12000000
100	10000000					97	9700000

GO08

Polydim-I			CTRL			CLR	
166	16600000		222	22200000		135	13500000
156	15600000		233	23300000		110	11000000
143	14300000					137	13700000
131	13100000					120	12000000

GO13

Polydim-I			CTRL			CLR	
146	14600000		294	29400000		95	9500000
157	15700000		219	21900000		105	10500000
123	12300000					75	7500000
136	13600000					107	10700000

GO18

Polydim-I			CTRL			CLR	
120	12000000		300	30000000		84	8400000
136	13600000		293	29300000		99	9900000
196	19600000					85	8500000
140	14000000					96	9600000

GO07

Polydim-I			CTRL			CLR	
95	9500000		156	15600000		95	9500000
87	8700000		222	22200000		135	13500000
97	9700000					137	13700000
cont	#VALUE!					98	9800000

CRM020

Polydim-I			CTRL			CLR	
166	16600000		279	27900000		86	8600000
156	15600000		265	26500000		93	9300000
143	14300000					91	9100000
131	13100000					94	9400000

ATCC14

Polydim-I			CTRL			CLR	
152	15200000		168	16800000		88	8800000
139	13900000		198	19800000		77	7700000
156	15600000					78	7800000
126	12600000					87	8700000
