# Supplementary material for: Antimycobacterial Activity of a New Peptide Polydim-I Isolated from Neotropical Social Wasp Polybia dimorpha
Source: PLoS One. 2016 Mar 1;11(3):e0149729. doi: 10.1371/journal.pone.0149729 (PMC4773228; doi:10.1371/journal.pone.0149729)
Supplement: S3 Fig — (PDF) [file pone.0149729.s003.pdf]

Polydim I — Cytotoxicity assays

LDH

Concentration (μg/ml)	cytotoxicity (%)		
7.6	7	0	3
15.2	0	5	2
60.8	16.7	15	20
121.6	70	67	75

Hemolysis

Concentration (μg/ml)	hemolysis (%)		
7.6	0	0.1	0.3
15.2	0	0.3	0.4
60.8	0	0.4	0.5
121.6	1.8	2.3	2.2
