# Supplementary material for: Antimycobacterial Activity of a New Peptide Polydim-I Isolated from Neotropical Social Wasp Polybia dimorpha
Source: PLoS One. 2016 Mar 1;11(3):e0149729. doi: 10.1371/journal.pone.0149729 (PMC4773228; doi:10.1371/journal.pone.0149729)
Supplement: S4 Fig — (PDF) [file pone.0149729.s004.pdf]

DATE: 09/30/2014 Experiment 1 In vivo

LUNG

PBS

**Day 1**			60	60000	10^2
			62	62000	
**Day 18**			91	910000	10^3
			93	930000	
**Day 26**			66	660000	10^3
			60	600000	
			50	500000	
			59	590000	

CLR 200mg

**Day 1**	60	600000	10^2
	62	620000	
**Day 18**	91	910000	10^3
	93	930000	
**Day 26**	10	1000	10^1
	15	1500	
	9	900	
	7	700	

Polydim-I 2mg

**Day 1**	60	600000	10^2
	62	620000	
**Day 18**	91	910000	10^3
	93	930000	
**Day 26**	100	10000	10^1
	135	13500	
	124	12400	
	158	15800	

Polydim-I 1mg

**Day 1**	60	600000	10^2
	62	620000	
**Day 18**	91	910000	10^3
	93	930000	
**Day 26**	11	11000	10^2
	28	28000	
	22	22000	
	18	18000	

Polydim-I 0.5mg

**Day 1**	60	600000	10^2
	62	620000	
**Day 18**	91	910000	10^3
	93	930000	
**Day 26**	122	122000	10^2
	108	108000	
	102	102000	
	127	127000	

MEDIA PBS 587500 (INFECTION 100%)

CLR 200mg

0.1702128	99.829787
0.2553191	99.744681
0.1531915	99.846809
0.1191489	99.880851

Polydim-I 2mg

1.7021277	98.297872
2.2978723	97.702128
2.1106383	97.889362
2.6893617	97.310638

Polydim-I 1mg

1.8723404	98.12766
4.7659574	95.234043
3.7446809	96.255319
3.0638298	96.93617

Polydim-I 0.5mg

20.765957	79.234043
18.382979	81.617021
17.361702	82.638298
21.617021	78.382979

SPLEEN

PBS

	70	700000	10^3
	72	720000	
	140	1400000	10^3
	141	1410000	
	122	1220000	10^3
	155	1550000	
	128	1280000	
	135	1350000	

CLR 200mg

**Day 1**	70	700000	10^3
	72	720000	
**Day 18**	140	1400000	10^3
	141	1410000	
**Day 26**	15	1500	10^1
	20	2000	
	5	500	
	7	700	

Polydim-I 2mg

**Day 1**	70	700000	10^3
	72	720000	
**Day 18**	140	1400000	10^3
	141	1410000	
**Day 26**	164	164000	10^2
	118	118000	
	118	118000	
	107	107000	

Polydim-I 1mg

**Day 1**	70	700000	10^3
	72	720000	
**Day 18**	140	1400000	10^3
	141	1410000	
**Day 26**	32	320000	10^3
	29	290000	
	40	400000	
	30	300000	

Polydim-I 0.5mg

**Day 1**	70	700000	10^3
	72	720000	
**Day 18**	140	1400000	10^3
	141	1410000	
**Day 26**	52	520000	10^3
	77	770000	
	81	810000	
	60	600000	

RESULTS DATE REDUCTION

MEDIA PBS 1350000 (INFECTION 100%)

CLR 200mg

0.1111111	99.888889
0.1481481	99.851852
0.037037	99.962963
0.0518519	99.948148

Polydim-I 2mg

12.148148	87.851852
8.7407407	91.259259
8.7407407	91.259259
7.9259259	92.074074

Polydim-I 1mg

23.703704	76.296296
21.481481	78.518519
29.62963	70.37037
22.222222	77.777778

Polydim-I 0.5mg

38.518519	61.481481
57.037037	42.962963
60	40
44.444444	55.555556

LIVER

PBS

	10	100000	10^3
	11	110000	
	110	1100000	10^3
	112	1120000	
	176	1760000	10^3
	165	1650000	
	156	1560000	
	155	1550000	

CLR 200mg

**Day 1**	10	100000	10^3
	11	110000	
**Day 18**	110	1100000	10^3
	112	1120000	
**Day 26**	51	5100	10^1
	35	3500	
	47	4700	
	53	5300	

Polydim-I 2mg

**Day 1**	10	100000	10^3
	11	110000	
**Day 18**	110	1100000	10^3
	112	1120000	
**Day 26**	70	700000	10^3
	85	850000	
	80	800000	
	97	970000	

Polydim-I 1mg

**Day 1**	10	100000	10^3
	11	110000	
**Day 18**	110	1100000	10^3
	112	1120000	
**Day 26**	118	1180000	10^3
	150	1500000	
	145	1450000	
	144	1440000	

Polydim-I 0.5mg

**Day 1**	10	100000	10^3
	11	110000	
**Day 18**	110	1100000	10^3
	112	1120000	
**Day 26**	130	1300000	10^3
	149	1490000	
	149	1490000	
	141	1410000	

MEDIA PBS 1630000 (INFECTION 100%)

CLR 200mg

0.3128834	99.687117
0.2147239	99.785276
0.2883436	99.711656
0.3251534	99.674847

Polydim-I 2mg

42.944785	57.055215
52.147239	47.852761
49.079755	50.920245
59.509202	40.490798

Polydim-I 1mg

72.392638	27.607362
92.02454	7.9754601
88.957055	11.042945
88.343558	11.656442

Polydim-I 0.5mg

79.754601	20.245399
91.411043	8.5889571
91.411043	8.5889571
86.503067	13.496933
