# Supplementary figures and images for: Antimycobacterial Activity of a New Peptide Polydim-I Isolated from Neotropical Social Wasp Polybia dimorpha
Source: PLoS One. 2016 Mar 1;11(3):e0149729. doi: 10.1371/journal.pone.0149729 (PMC4773228; doi:10.1371/journal.pone.0149729)

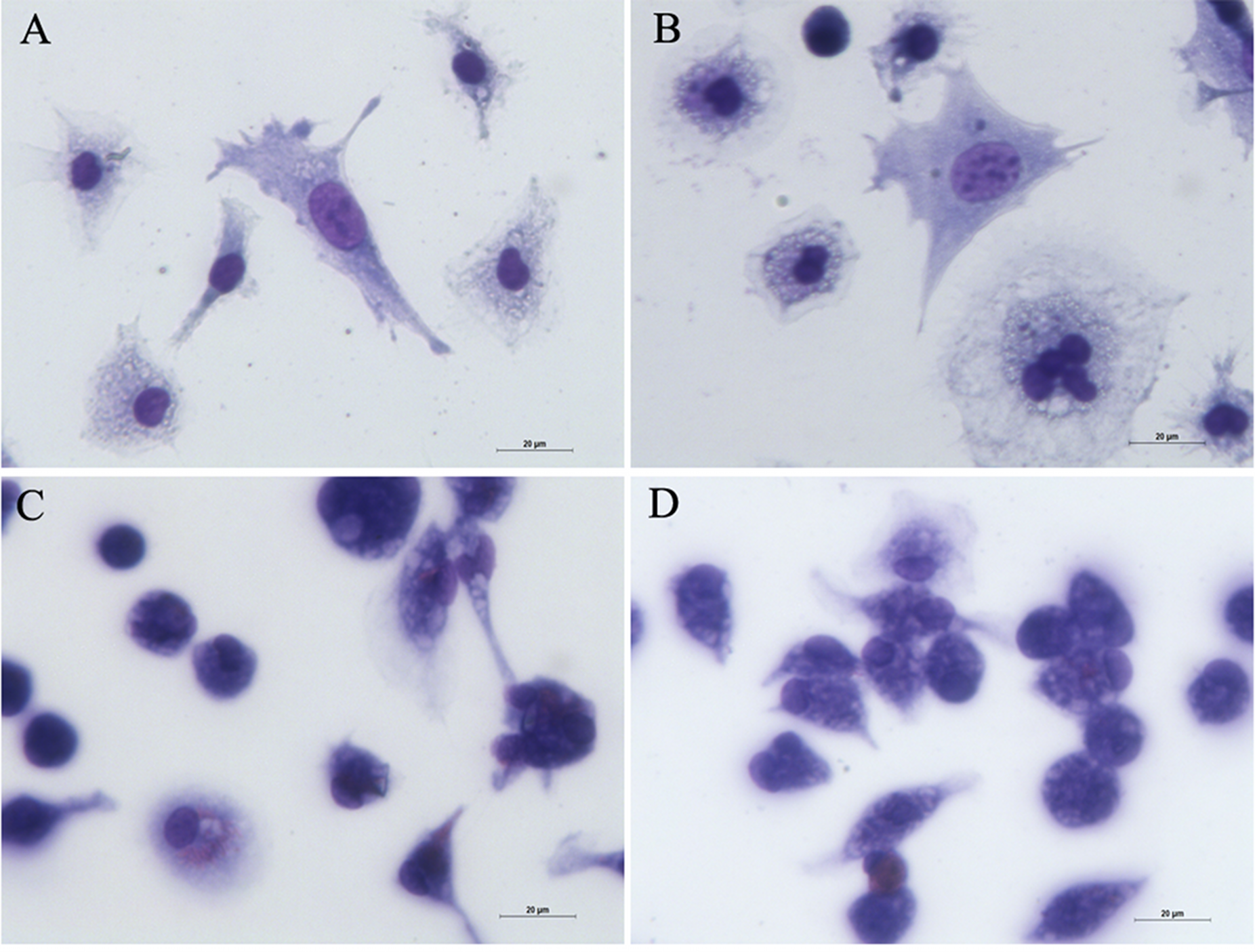

Supplement: S5 Fig — Macrophage morphology after Polydim-I treatment and infection. Instant-Prov staining of: (A) Untreated macrophages; (B) Polydim-I peptide-treated macrophages; (C) macrophages infected with Mycobacterium abscessus subsp. massiliense isolate GO 06 (MOI 10:1); and (D) macrophages infected with M. abscessus subsp. massiliense isolate GO 06 and treated with Polydim-I peptide. (TIF) [file pone.0149729.s005.tif]

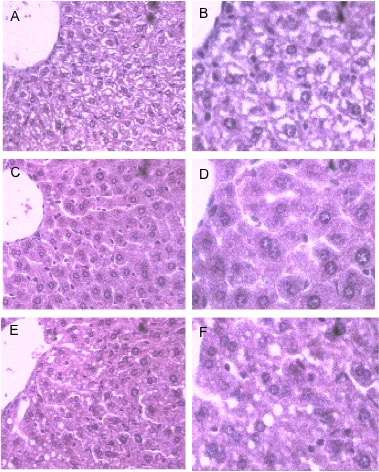

Supplement: S6 Fig — BALB/c mice were treated with 2 mg/kg/mLW (C and D) or 20 mg/kg/mLW (E and F) and 48 hours later the livers were processed and stained with HE. As controls, mice were treated with PBS (A and B). The results are presented at 40 x and 100 x magnification. (TIF) [file pone.0149729.s006.tif]
